# Supplementary material for: A hybrid CNN-XLSTM-GRU deep learning model with autoencoder-based feature selection for hypothyroidism diagnosis
Source: PLoS One. 2026 Apr 21;21(4):e0347203. doi: 10.1371/journal.pone.0347203 (PMC13099102; doi:10.1371/journal.pone.0347203)
Supplement: Attachment — Submitted filename: comments.docx [file pone.0347203.s001.docx]

The article was reviewed under the title " A Hybrid CNN-XLSTM-GRU Deep Learning Model with Autoencoder-Based Feature Selection for Hypothyroidism Diagnosis ".

Overall, the focus of the study is good. However, I would like to offer several recommendations that authors may find useful in the process of revising their manuscript:

1- Based on Figure 1 and the text of the article, it is stated that in the data preparation section, missing values were filled with mean and mode. Was there a specific reason for this or was it done simply based on the similarity of the data?
2- The order of some figures does not match the text of the article in terms of pages, which requires sorting the images for better coherence of the article.
3- In the table related to hyperparameters, the value of epochs is declared as 50, but in the text of the article and in the section Performance outcomes with deep learning models, this value is stated as 200, and even in Figure 11 it is stated as 250. Please review these discrepancies.
4- Regarding comment number 3, it is not clear whether the model was tested in different epochs or whether the early stopping method was used.
5- The resolution of Figures 8 and 10 is low in terms of row and column headings, so it is better to use higher quality figures.
6- Also, the resolution of Figure 12 is low. Considering that it is stated in the section “Error Analysis and Model Robustness” that “Furthermore, Figure 12 shows that the FusionNet-CXG model demonstrated significant…….”, it is better for the resolution of the figure to be higher.
