# Supplementary material for: A hybrid CNN-XLSTM-GRU deep learning model with autoencoder-based feature selection for hypothyroidism diagnosis
Source: PLoS One. 2026 Apr 21;21(4):e0347203. doi: 10.1371/journal.pone.0347203 (PMC13099102; doi:10.1371/journal.pone.0347203)
Supplement: Attachment — Submitted filename: Response to Reviewer.docx [file pone.0347203.s004.docx]

**Journal**	: PLOS ONE

**Manuscript Number	:** PONE-D-25-37245

**Type**	: Research article

**Title**	: A Hybrid CNN-XLSTM-GRU Deep Learning Model with	Autoencoder-Based Feature Selection for Hypothyroidism Diagnosis

Dear Editor,

Thank you for your useful comments and suggestions for the improvement of our manuscript. We have modified the manuscript accordingly, and detailed corrections are listed below for each comment from the reviewers.

1) The reviewer's comments are highlighted in black colour.

2) Our responses to the comments are highlighted in blue colour.

**Response to Editor**

**Comment 1:** Experimental setup (including data preprocessing and results postprocessing) should be described clearly and with more details.

**Response 1:** The authors thank the editor for the suggestion. The manuscript has been updated to include a clearer description of the experimental setup, covering both data preprocessing and results post-processing. In addition, **Tables 4, 5, and 6 in the Results section now provide a structured summary of the experimental analysis**, with Table 4 outlining the end-to-end pipeline, Table 5 presenting the computational environment, and Table 6 reporting the detailed performance evaluation.

**Comment 2:** Analysis of measurement errors and statistical properties of the results should be included.

**Response 2:**	The authors thank the editor for the valuable comment. We updated the Results to include statistical analysis: for each metric, we report fold-wise dispersion, mean ± SD, 95% CIs, and median/IQR/min–max; we also quantify variability across CV splits and flag outlier folds—addressing measurement error and distributional properties and strengthening reliability and reproducibility.

**Comment 3:** Language problems should be removed.

**Response 3:**	The authors thank the editor for the valuable comment. We thoroughly updated the manuscript for clarity, grammar, and style, aligning wording with the journal’s preferred English variety.

**Comment 4:** Figures should have all axes described precisely (also with values - see problems with Fig. 2).

**Response 4:**	The authors sincerely thank the editor for the valuable feedback. The manuscript has been carefully revised, and all figures have been corrected to ensure that axes are fully labeled with precise descriptions, values, and units.

**Comment 5:** Figures should be clearly readable (see problems with Fig. 6, Fig. 8, Fig. 10, Fig. 12, Fig. 13).

**Response 5:**	The authors thank the editor for this observation. All figures have been updated to ensure readability. Specifically, Figures 6, 8, 10, 12, and 13 were regenerated at high resolution (>300 dpi), font sizes increased, and colour contrasts improved. Axes, legends, and annotations have been standardised to maintain consistency and clarity across the manuscript.

**Comment 6:** Equations should be presented and described properly (see: (9)(11), what are b_h, g_z?),

**Response 6:**	The authors thank the editor for pointing this out. We have carefully revised the equations to ensure they are properly formatted and described in detail. Specifically, equations (9) and (11) have been retypeset with consistent mathematical notation, and all parameters and symbols are now explicitly defined in the text immediately after their first appearance. For example, $b_{h}$is described as the bias term for the hidden state, while $g_{z}$denotes the update gate activation in the GRU cell. These clarifications are now included in the revised manuscript.

**Comment 7:** Format of references should be uniform (see. [18], [23]),

**Response 7:**	The authors thank the editor for the valuable comment. In the updated manuscript, we format of all in-text citations and reference entries has been standardised according to the journal guidelines.

**Comment 8:** Authors should consider more related works which are not from India and China,

**Response 8:**	The authors thank the editor for the valuable comment. In the updated manuscript, we format of all in-text citations and reference entries has been standardised according to the journal guidelines.

**Comment 9:** Discrepancies between numbers of training epochs reported in the text, in tables and in figures should be clarified,

**Response 9:**	The authors thank the editor for the valuable comment. In the updated manuscript, we have reconciled all epoch counts. Text, tables, and figures now follow a single policy: training ran up to 500 epochs with EarlyStopping on validation loss (patience 8–20), and results are taken from the best epoch per run. Tables report performance at the best epoch; figures showing training curves display the full trajectory but mark the best epoch. We also added the median [IQR] best epoch across runs to Table 6 and a caption note to clarify this.

**Comment 10:** Comparison with related results from other Authors would greatly improve the value of the manuscript.

**Response 10:** The authors thank the editor for this valuable suggestion. The updated manuscript now includes an expanded **Related Works and Results discussion** where we compare our findings against other state-of-the-art studies. Specifically, we contrast FusionNet-CXG performance with recent hybrid deep learning ensembles for disease classification (e.g., CNN–LSTM–MLP for diabetes diagnosis, CNN–BiLSTM/BiGRU ensembles for heart disease, attention-based CNN–LSTM for COVID-19 cough analysis, hybrid ensemble for Alzheimer’s). This contextualizes our contribution within a broader global literature and highlights both performance gains and methodological differences.

**Response to Reviewer #1 Comments**

**Comment 1:** The model achieves high accuracy, there is limited discussion of interpretability tools to help clinicians understand decision-making.

**Response 1:** The authors thank the reviewer for their valuable and encouraging comment which has significantly contributed to enhancing the clarity and overall quality of the paper. The SHAP (interpretability tool) has been incorporated into the revised manuscript in **Results section.**

**Comment 2:** Despite the use of autoencoders, the paper would benefit from more rigorous reporting on overfitting.

**Response 2:**	The authors thank the reviewer for their valuable comment. We performed a leakage-free 5×10 Repeated Stratified Cross-Validation protocol in which the autoencoder, feature scalers, and SMOTE-NC were fit strictly within each fold’s training split. Models were trained with early stopping under a 500-epoch cap. We also provide training-vs-validation learning curves for the proposed model and baselines, which show close alignment and no late-epoch divergence, indicating minimal overfitting.

**Comment 3:** The work reliability is poor as it hasn't been performed in repeated cross-validation testing.

**Response 3:** We express our heartfelt gratitude to the reviewer for this significant recommendation. In the revised manuscript in the **Results** **section**, we have now evaluated the model using repeated stratified k-fold cross-validation (5 folds × 10 repeats = 50 runs) with full leakage control (all preprocessing, SMOTE-NC, autoencoder, and classifier fit only on the training split; early stopping on an internal validation subset). We report accuracy, specificity (TNR), **precision, recall, and F1-score**, summarised across 50 runs as mean ± SD with 95% CIs. To further evidence reliability, we quantify train–val generalisation gaps at the best validation epoch, show the distribution of best epochs from early stopping, and include a calibration curve with Brier score from pooled out-of-fold predictions. Overall, these additions demonstrate stable and well-calibrated performance.

**Response to Reviewer #2 Comments**

**Comment 1:** The motivation of the paper is not clear.

**Response 1:** the authors thank the reviewer for the helpful suggestion. We've added a short "Motivation" paragraph at the end of the **Introduction** that explains the clinical need for reliable, understandable early detection of hypothyroidism, points out key methodological gaps (imbalance, noisy/redundant features, limited temporal/spatial modelling), and explains why our FusionNet-CXG approach (with SMOTE-NC, an autoencoder bottleneck, and SHAP) is the best way to meet that need. We think this change makes the paper's purpose clear and fits well with what we've added.

**Comment 2:** Contributions are not mentioned. Most importantly, the structure of the Introduction section is very poor.

**Response 2:** The authors thank the reviewer for the helpful feedback. In the revised manuscript, we have restructured the **Introduction section** for clarity and now include a dedicated Contributions paragraph.

**Comment 3**: Related schemes are not discussed properly. The following references may be cited:

- A Smart Healthcare System Using Consumer Electronics and Federated Learning to Automatically Diagnose Diabetic Foot Ulcers
- Drug Target Interaction Prediction Using Machine Learning Techniques – A Review
- HCNNet: hybrid convolution neural network for automatic identification of ischaemia in diabetic foot ulcer wounds
- Analysis and Prediction of COVID-19 Using Growth Analysis Models: A Case Study
- Role of internet of things and machine learning in smart healthcare
- Early prediction of cognitive impairments using physiological signal for enhanced socioeconomic status

**Response 3:** We sincerely thank the reviewer for the valuable recommendation. In the revised manuscript, we have extended the Related Work section to address important methods and incorporated citations for the recommended publications.

**Comment 4:** The proposed scheme is unstructured. It is hard to identify the novelty of the proposed work.

**Response 4:** The authors thank the reviewer for the helpful observation. In the revised manuscript, we added a concise workflow schematic (Fig. 1) with an overview paragraph, a clearly labelled Contributions list in the Introduction, and reorganized Methods into logical blocks data, preprocessing, class balancing (SMOTE-NC), autoencoder feature extraction, FusionNet-CXG, and training/evaluation, so the workflow is clear. Our novelty lies in a leakage-free AE + SMOTE-NC pipeline and a hybrid CNN–XLSTM–GRU classifier, validated with 5×10 repeated CV, per-fold statistical tests, and SHAP, yielding state-of-the-art accuracy with clinically consistent explanations.

**Comment 5:** Equations and figures are not represented properly. Also, the key terms of many equations are not defined.

**Response 5:** We thank the reviewer for these insightful suggestions. In response, we have updated the manuscript to (i) standardise all equations and numbering, (ii) define every symbol on first usage and (iii) reformat pictures with clear panel labels, scale bars, and self-contained captions. These revisions enhance clarity and ensure that all essential terminology is clearly defined and uniformly applied throughout.

**Comment 6:** Technical discussion on results is not given. Moreover, the results are not convincing.

**Response 6:** We thank the reviewer for the helpful comment. In revision, we report cross-validated mean±SD and 95% CIs for all metrics and conduct paired per-fold significance tests with effect sizes against baselines. We also include representative training–validation learning curves and link SHAP attributions to hypothyroid physiology, which together strengthen the credibility of the results.

**Comment 7:** What is the use Fig. 12? It is not properly visible.

**Response 7:** We thank the reviewer for the comment. Figure 12 shows Autoencoder (AE) latent dimensions align with thyroid-function indicators, preserving clinical structure and reducing redundancy thereby justifying AE features in FusionNet-CXG. We provide a high-resolution (≥600 dpi), clearly labelled, hierarchically clustered version.

**Comment 8:** The English language is very poor.

**Response 8:** We thank the reviewer for the comment. We have thoroughly revised the manuscript to improve the English, correcting grammar and punctuation, simplifying lengthy sentences, standardizing terminology/abbreviations, and polishing figure/table captions.

**Comment 9:** The organization of the paper is poor. For example, the authors should delete the Study Limitations section. The limitations can be mentioned in the last section. The authors must go through some reputed journal papers before addressing these comments.

**Response 9:** We thank the reviewer for the guidance. We have **removed the standalone “Study Limitations” section** and placed the limitations in the **final paragraph of the Discussion**.

**Comment 10:** Important references are missing, and all the details of the references are not given.

**Response 10:** The authors thank the reviewer for the comment. We have added the missing key references and completed all citation details (authors, titles, journal, year, volume/issue, pages, DOIs). Status of Goitre or Thyroid Disorders in India,” 2022, [Online]. Available: <https://pib.gov.in/PressReleasePage.aspx?PRID=1796440>, and Kaggle, “Hypothyroid dataset.” [Online]. Available: <https://www.kaggle.com/datasets/kumar012/hypothyroid> are not journal articles, so we could not give citation details.

**Comment 11:** Don't include the designation of the author.

**Response 11:** We express our heartfelt gratitude to the reviewer for these perceptive and progressive recommendations. We have removed all author designations and kept only names and affiliations.

**Response to Reviewer #3 Comments**

**Comment 1:** Based on Figure 1 and the text of the article, it is stated that in the data preparation section, missing values were filled with mean and mode. Was there a specific reason for this or was it done simply based on the similarity of the data?

**Response 1:** The authors thank the reviewer's comment. There was a specific reason, not a similarity-based choice. After checking distributions, the z-scored continuous labs (TSH, TT4, T3, FTI, T4U) were roughly symmetric, so we used mean imputation (low bias, minimises squared error); for categorical variables we used mode imputation to preserve level frequencies needed by SMOTE-NC. All imputers were fit within each training fold and applied to its validation split to keep the pipeline leakage-free.

**Comment 2:** The order of some figures does not match the text of the article in terms of pages, which requires sorting the images for better coherence of the article.

**Response 2:** The authors thank the reviewer for the observation. In the revised manuscript we reordered and renumbered all figures to follow their first mention in the text, updated captions.

**Comment 3:** In the table related to hyperparameters, the value of epochs is declared as 50, but in the text of the article and in the section Performance outcomes with deep learning models, this value is stated as 200, and even in Figure 11 it is stated as 250. Please review these discrepancies.

**Response 3:** The authors express gratitude to the reviewer for pointing this out. We have standardised the training setup across the paper: models were trained with a 500-epoch cap and early stopping (monitor=val_loss, patience=10, restore_best_weights=True). Effective training stopped earlier depending on the fold. We revised the manuscript updating the hyperparameter table, Figure, and the “Performance outcomes” text to remove the conflicting 50/200/250 values and reflect this single, consistent training setup.

**Comment 4:** Regarding comment number 3, it is not clear whether the model was tested in different epochs or whether the early stopping method was used.

**Response 4:** The authors thank the reviewer for pointing this out. We used early stopping in all experiments (with a 500-epoch cap); training stopped automatically based on validation loss.

**Comment 5:** The resolution of Figures 8 and 10 is low in terms of row and column headings, so it is better to use higher quality figures.

**Response 5:** The authors thank the reviewer for this comment. In the updated manuscript, we have replaced **Figures 8 and 10** with **high-resolution** versions, increased the row/column label font sizes, and updated the manuscript with the revised figures and captions for clear readability.

**Comment 6:** Also, the resolution of Figure 12 is low. Considering that it is stated in the section “Error Analysis and Model Robustness” that “Furthermore, Figure 12 shows that the FusionNet-CXG model demonstrated significant…….”, it is better for the resolution of the figure to be higher.

**Response 6:** The authors thank the reviewer for this helpful observation. In the updated manuscript, we have replaced **Figure 12** with a higher-resolution version to improve readability. In addition, we corrected the in-text reference in the “**Error Analysis and Model Robustness**” section: the sentence “Furthermore, Figure 12 shows that the FusionNet-CXG model demonstrated significant…….”has been revised to “Furthermore, Figure 15 shows that the FusionNet-CXG model demonstrated significant…….”

**Response to Reviewer #4 Comments**

**Comment 1:** The paper is well-written with good quality to meet the criteria of publishing after the improvements for research reproducibility. The authors should add the pseudocode for the proposed approach and the statistical analysis for the results obtained. I recommend Rcommander in Rstudio as a helper tool for that.

**Response 1:** The authors thank the reviewer for the helpful suggestions. In response, we have implemented all requested improvements and updated the manuscript. Specifically, we have added the pseudocode for the proposed FusionNet-CXG approach and incorporated comprehensive statistical analyses of the obtained results in **Materials and Methods, Results section**. In particular, we used 5×10 Repeated Stratified CV (50 runs) and report all metrics as mean ± SD with 95% bootstrap confidence intervals. We also conducted paired *t*-tests (p-values reported) for pairwise comparisons.
