# Supplementary material for: A hybrid CNN-XLSTM-GRU deep learning model with autoencoder-based feature selection for hypothyroidism diagnosis
Source: PLoS One. 2026 Apr 21;21(4):e0347203. doi: 10.1371/journal.pone.0347203 (PMC13099102; doi:10.1371/journal.pone.0347203)
Supplement: Attachment — Submitted filename: comments.docx [file pone.0347203.s003.docx]

The article was reviewed under the title "A Hybrid CNN-XLSTM-GRU Deep Learning Model with Autoencoder-Based Feature Selection for Hypothyroidism Diagnosis".

Overall, the focus of the study is good. However, I would like to offer several recommendations that authors may find useful in the process of revising their manuscript:

1- Despite the comments of previous respected reviewers that the images do not match the text of the article, this problem has still not been resolved.
2- Also, the quality of the images, including images 8, 9,12 and 13, is still low.
3- Given that the dataset is from 1987 and that preprocessing was performed on the data, especially for missing values, and given the novelty of the topic of the article, was there a specific reason for not using the new dataset?
4- In the comparison table (Table 11), do the collections of articles selected for comparison match the data set of the article? For example, the data set used in references 21 and 61 is different from the data set of the article. To demonstrate the superiority of the model, it is better to compare the results with other results on the said data set.
