# Supplementary material for: A hybrid CNN-XLSTM-GRU deep learning model with autoencoder-based feature selection for hypothyroidism diagnosis
Source: PLoS One. 2026 Apr 21;21(4):e0347203. doi: 10.1371/journal.pone.0347203 (PMC13099102; doi:10.1371/journal.pone.0347203)
Supplement: Attachment — Submitted filename: PlosOne-Response-II.docx [file pone.0347203.s006.docx]

**Journal**	: PLOS ONE

**Manuscript Number	:** PONE-D-25-37245R1

**Type**	: Research article

**Title**	: A Hybrid CNN-XLSTM-GRU Deep Learning Model with Autoencoder-Based Feature Selection for Hypothyroidism Diagnosis

Dear Editor,

Thank you for noting that the manuscript was reviewed under the title “A Hybrid CNN-XLSTM-GRU Deep Learning Model with Autoencoder-Based Feature Selection for Hypothyroidism Diagnosis.” We thank the reviewer for their recommendations and have revised the manuscript accordingly. Detailed corrections are provided below for each of the reviewer’s comments.

1) The reviewer's comments are highlighted in black colour.

2) Our responses to the comments are highlighted in blue colour.

**Response to Reviewer Comments**

**Comment 1:** Despite the comments of previous respected reviewers that the images do not match the text of the article, this problem has still not been resolved.

**Response 1:**	The authors sincerely thank the reviewer for this observation. We fully acknowledge the earlier concern raised regarding mismatches between images and the corresponding text. In this revised version of the manuscript, all figure numbers, captions, and in-text references have been carefully verified and corrected. Specifically, the previous inconsistency involving Figures 5 and 6 has now been fully resolved in the **Data preprocessing section (Page 8)** by assigning each figure a unique and accurate reference that aligns with its description in the text. We rechecked the entire manuscript to ensure that every image now correctly matches its caption and all textual references, and no discrepancies remain.

**Comment 2:** Also, the quality of the images, including images 8, 9,12 and 13, is still low.

**Response 2:** The authors thank the reviewer for highlighting this issue. We acknowledge that the earlier version contained low-resolution images, particularly Figures 8, 9, 12, and 13. In this revised submission, all affected figures have been regenerated at high resolution (400 DPI).

**Comment 3:** Given that the dataset is from 1987 and that preprocessing was performed on the data, especially for missing values, and given the novelty of the topic of the article, was there a specific reason for not using the new dataset?

**Response 3:** The authors thank the reviewer for this valuable comment. We used the 1987 hypothyroid dataset because it is the only publicly available, feature-rich clinical dataset that includes both numerical hormone tests and detailed categorical medical attributes required for hybrid deep-learning models. Although older, it remains the standard benchmark in recent thyroid-prediction studies, enabling fair comparison with state-of-the-art work. Newer real-world datasets were not accessible due to privacy restrictions and a lack of open clinical data, but we mitigated dataset limitations through rigorous preprocessing, imputation, class balancing, and autoencoder-based denoising. Future work will incorporate modern clinical datasets once ethically accessible.

**Comment 4:** In the comparison table (Table 11), do the collections of articles selected for comparison match the data set of the article? For example, the data set used in references 21 and 61 is different from the data set of the article. To demonstrate the superiority of the model, it is better to compare the results with other results on the said data set.

**Response 4:** The authors thank the reviewer for this important observation. We fully understand the concern that comparing results obtained from different datasets can lead to misleading performance conclusions. In response, we carefully re-examined all studies listed in Table 11 and confirmed that some cited works (e.g., references [21] and [60] (*61)) indeed used datasets that differ from the hypothyroid dataset employed in our study.

To make sure everything is fair and clear, we made the following changes to the manuscript:

- Clarification added: The wording after Table 11 has been changed to make it clear which studies utilised the same hypothyroid dataset and which studies used distinct datasets.
- Controlled comparisons: The baseline models (CNN + LSTM and CNN + BiLSTM) were re-implemented and tested on the same hypothyroid dataset, with the same parameters for preprocessing, balancing, and validation. The same-dataset findings show that the proposed FusionNet-CXG model works better.
- Table note added: A footnote was added to Table 11 to show that research utilising different datasets are marked with a dagger symbol (†) and to make it clear that those entries are only there for context and not for direct numerical comparison.

These changes make sure that all performance comparisons are now clear and that claims of superiority are based on findings from the same dataset. The new explanation can be found on page 29, lines 1–23 of the amended manuscript.
