# Supplementary material for: A hybrid CNN-XLSTM-GRU deep learning model with autoencoder-based feature selection for hypothyroidism diagnosis
Source: PLoS One. 2026 Apr 21;21(4):e0347203. doi: 10.1371/journal.pone.0347203 (PMC13099102; doi:10.1371/journal.pone.0347203)
Supplement: Attachment — Submitted filename: Reviewer Response-II.docx [file pone.0347203.s007.docx]

**Journal**	: PLOS ONE

**Manuscript Number	:** PONE-D-25-37245R2

**Type**	: Research article

**Title**	: A Hybrid CNN-XLSTM-GRU Deep Learning Model with	Autoencoder-Based Feature Selection for Hypothyroidism Diagnosis

Dear Editor,

Thank you for your useful comments and suggestions for the improvement of our manuscript. We have modified the manuscript accordingly, and detailed corrections are listed below for each comment from the reviewers.

1) The reviewer's (Editor) comments are highlighted in black colour.

2) Our responses to the comments are highlighted in blue colour.

**Response to Editor**

**Comment 1:** Fig 6.: values are unreadable. This should not happen. Presentation of such figure has very limited value, and decreases the overall quality of the manuscript.

**Response 1:** The authors sincerely thank the author for highlighting this important issue. We agree that the readability of Fig. 6 in the original submission was inadequate. To address this concern, Fig. 6 has been completely revised by increasing the font size of all labels and values, improving resolution, and enhancing visual clarity. The updated figure now ensures that all numerical values and annotations are clearly readable. These changes significantly improve the presentation quality and interpretability of the figure.

**Comment 2:** Table 5 repeats multiple information from Table 4. Is this needed?

**Response 2:**	The authors thank the editor for the valuable comment. We agree that Table 5 overlapped with Table 4 in the original manuscript (mainly software/framework details). We have revised Table 4 to cover only the experimental configuration and modelling pipeline, and we have trimmed Table 5 to include only the software and hardware environment for reproducibility. The overlap has been removed to avoid repetition and improve readability.

**Comment 3:** Language Table 5 includes elements such as "Storage: Google Drive", "Operating System: Google Colab Environment". Are results influenced by those elements (e.g by the type of data storage)? If yes it should be discussed. If no, then not needed information should be removed. Btw. Google Colab Environment is not an Operating System (typically it uses some form of Linux as an OS).

**Response 3:**	The authors thank the editor for the comment. The experimental results are not influenced by the data storage type or execution platform. Therefore, unnecessary information such as storage details has been removed. We have also corrected the operating system description to Linux (Ubuntu, Google Colab default). Table 5 now includes only essential software and hardware details relevant to reproducibility.

**Comment 4:** Fig 9, Fig 10b, Fig 11, Fig 12b, Fig 13, Fig 14b, Fig 15: vertical axis, description of the axis should present units [% or 1? it is not clear if 0.918 means 0.918% or 91,8%]

**Response 4:**	The authors sincerely thank the editor for highlighting this issue. The vertical axis values represent performance metrics expressed in percentage. To remove any ambiguity, all affected figures (Figs. 9, 10b, 11, 12b, 13, 14b, and 15) have been revised to explicitly indicate the unit [%] in the y-axis labels and figure captions.

**Comment 5:** Fig 9, Fig 11, Fig 13, Fig 15, Fig 16, Table 9, Table 10: title is not precise; it is not written if presented result is for training or testing data.

**Response 5:**	The authors thank the editor for this comment. All results presented in Fig. 9, Fig. 11, Fig. 13, Fig. 15, Fig. 16, Table 9, and Table 10 correspond to testing data. To ensure clarity, the titles and captions of the mentioned figures and tables have been revised to explicitly state that the reported results are based on testing performance.

**Comment 6:** Fig 9, Fig 11, Fig 13, Fig 15: Error whiskers or regions should be also presented within those figures.

**Response 6:**	The authors thank the editor for this suggestion. Error whiskers representing variability (95% confidence intervals) have now been included in Fig. 9, Fig. 11, Fig. 13, and Fig. 15 to provide a clearer representation of result dispersion.

**Comment 7:** Table 8: "Â" character before "±" seems to be improper (all rows including the header)

**Response 7:**	The authors thank the editor for this suggestion. The ‘Â’ character was an encoding artifact introduced during table formatting; it has been removed and the ‘±’ symbol is now rendered correctly throughout Table 8.

**Comment 8**: Fig 2, Fig 4, Fig 15- Fig 17: There is no need to repeat title above the figure. Title below the figure should be enough.

**Response 8:**	The authors thank the editor for the valuable comment. In the updated manuscript, the repeated titles above the figures have been removed, and only the figure captions below the figures are now retained, as suggested.

**Comment 9:** Table 11. It would be more clear if "Note: Entries marked with (†) correspond to studies that used datasets different from the UCI/Kaggle hypothyroid dataset employed in this work." was placed just under the table (e.g. as a footnote) or within its title.

**Response 9:**	The authors thank the editor for the suggestion. The note has been moved and is now placed directly below Table 11 as a footnote in the updated manuscript, improving clarity and readability.

**Comment 10**: Performed comparison of various models is not based on statistical tests. This limits the confidence of presented analysis and conclusions. It should be discussed as an limitation of the study.

**Response 10:** The authors thank the editor for this important observation. We agree that the comparison of models is not supported by formal statistical significance tests, which may limit the strength of the conclusions. This limitation has now been explicitly acknowledged and discussed in the Discussion section’s last paragraph of the revised manuscript.
