# Supplementary material for: A hybrid CNN-XLSTM-GRU deep learning model with autoencoder-based feature selection for hypothyroidism diagnosis
Source: PLoS One. 2026 Apr 21;21(4):e0347203. doi: 10.1371/journal.pone.0347203 (PMC13099102; doi:10.1371/journal.pone.0347203)
Supplement: Attachment — Submitted filename: Response-IV.docx [file pone.0347203.s008.docx]

**Journal**	: PLOS ONE

**Manuscript Number	:** PONE-D-25-37245R3

**Type**	: Research article

**Title**	: A Hybrid CNN-XLSTM-GRU Deep Learning Model with Autoencoder-Based Feature Selection for Hypothyroidism Diagnosis

Dear Editor,

Thank you for noting that the manuscript was reviewed under the title “A Hybrid CNN-XLSTM-GRU Deep Learning Model with Autoencoder-Based Feature Selection for Hypothyroidism Diagnosis.” We thank the reviewer for their recommendations and have revised the manuscript accordingly. Detailed corrections are provided below for each of the reviewer’s comments.

1) The reviewer's comments are highlighted in black colour.

2) Our responses to the comments are highlighted in blue colour.

**Response to Reviewer Comments**

**Comment 1:** Language problems:

1.1 "Precision refers to all positively labelled items. recall refers to a positive(hypothyroid) class forecast."
=> "Precision refers to all positively labelled items. Recall refers to a positive(hypothyroid) class forecast."

1.2 "The Mathematical formulae for various statistical methods used in model assessment are delineated in Eqs (15-20)."=> "The mathematical formulae for various statistical methods used in model assessment are delineated in Eqs (15-20)."

**Response 1:**	The authors thank the reviewer for these helpful language corrections. Both sentences have been updated in the manuscript.

**Comment 2.1:** Fig 2: vertical axis: are Authors sure that "Frequency" is the right description. Please consider "Number of subjects" or "Count".

**Response 2.1:** The authors thank the reviewer for highlighting this issue. We agree that “Frequency” was not the clearest label for the histogram y-axis. Since Fig. 2 displays raw bin counts, the vertical axis label has been revised to “Count.” The figure description was also checked and remains consistent with this change.

**Comment 2.2:** Fig 15 - blue blocks are not translucent and hide other information

**Response 2.2:** The authors thank the reviewer for this valuable comment. Figure 15 has been revised to improve readability. The blue confidence-interval regions are now shown with transparency, so they no longer obscure the other plotted elements. The overall accuracy reference line remains clearly visible in the revised figure.

**Comment 2.3:** Fig 10b, Fig 12b, Fig 14b: Vertical axis (accuracy) should present units (0.9 can mean 90% as well as 0.9%)

**Response 2.3:** The authors thank the reviewer for this important observation. We have updated the vertical axis labels in Fig. 10(b), Fig. 12(b), and Fig. 14(b) to “Accuracy (%)” so that the unit is explicitly stated and the values cannot be misinterpreted.

**Comment 2.4:** Fig 9, Fig 11, Fig 13: axis descriptions are of low quality. Please consider changing figure to vector form. Also, the legend entry "95% CI whiskers" is redundant (and not needed).

**Response 2.4:** We thank the reviewer for this helpful comment. We have revised Fig. 9, Fig. 11, and Fig. 13 to improve the clarity of the axis descriptions. The figures were also replaced with higher-quality versions to improve readability, and the redundant legend entry “95% CI whiskers” has been removed.

**Comment 2.5:** Fig 16: The title is too general. Is it done on training or testing data
Also: Error whiskers overlap with some values (this limits readability). Please move the values.

**Response 2.5:** The authors thank the reviewer for this helpful comment. We agree that the original title of Fig. 16 was too general. The figure title/caption has been revised to clarify that the comparison is based on the test-fold performance obtained under 10 repetitions of 5-fold stratified cross-validation. In addition, the value labels were repositioned so that they no longer overlap with the error whiskers, thereby improving figure readability.

**Comment 2.6:** Table 9: estimated measurement errors should be presented

**Response 2.6:** The authors thank the reviewer for the suggestion. Table 9 has been revised to include estimated performance variability for all reported metrics. Specifically, each metric is now presented as mean ± standard deviation, together with the corresponding 95% confidence interval, based on the repeated stratified cross-validation runs. This revision provides a clearer indication of the variation in model performance across repeated evaluations.

**Comment 2.7:** numbering of equations: (8), (14) are improperly justified (should be: to the right)

**Response 2.7:** The authors thank the reviewer for pointing this out. The formatting of Eqs. (8) and (14) has been corrected, and the equation numbers are now properly aligned to the right in the updated manuscript.

**Comment 3.1:** Table 10: Authors are using paired t-test. It is not clear if t-test assumptions were verified (distributions normality and similarity of variances)?

**Response 3.1:** The authors thank the reviewer for this comment. We have clarified the statistical procedure used in Table 10. The paired t-test was performed on matched repetition-level mean accuracy values obtained from the repeated cross-validation runs. Since the test was based on paired observations, equality of variances between independent groups was not required. This clarification has been added to the revised manuscript.

**Comment 3.2:** It is not clear what 5x10 fold CV means: is it 10 times repeated 5-fold CV or 5 times repeated 10-fold CV?

**Response 3.2:** The authors thank the reviewer for this comment. In this study, 5×10-fold cross-validation refers to 10 repetitions of 5-fold stratified cross-validation, resulting in a total of 50 runs. This has been clarified in the revised manuscript.

**Comment 3.3:** It is not clear how the t-test was performed. Treating separate folds as data blocks in statistical test is not a good solution as folds are statistically not independent. Folds should be averaged and repeated CV should be used to multiply data blocks.

**Response 3.3:** The authors thank the reviewer for this important comment. We agree that the individual folds from the same repeated cross-validation procedure should not be treated as statistically independent. Therefore, the five-fold-wise accuracy values within each repetition were first averaged to obtain one repetition-level mean accuracy. Since the evaluation used 10 repetitions of 5-fold stratified cross-validation, this resulted in 10 paired repetition-level accuracy values for each model comparison. The paired t-test was then performed on these 10 repetition-level means. This clarification has been added to the revised manuscript.

**Comment 3.4:** It is not clear how the averaging of classification accuracy was done - was it micro or macro averaging?

**Response 3.4:** The authors thank the reviewer for the comment. Accuracy was not computed using micro- or macro-averaging. Instead, 10 repetitions of 5-fold stratified cross-validation were performed. In each repetition, accuracy was calculated on each of the five test folds, and the resulting five-fold-wise accuracy values were averaged to obtain one repetition-level mean accuracy. The final reported accuracy was then obtained by averaging these 10 repetition-level mean accuracies. This clarification has been added to the revised manuscript.
