# Supplementary material for: A hybrid CNN-XLSTM-GRU deep learning model with autoencoder-based feature selection for hypothyroidism diagnosis
Source: PLoS One. 2026 Apr 21;21(4):e0347203. doi: 10.1371/journal.pone.0347203 (PMC13099102; doi:10.1371/journal.pone.0347203)
Supplement: Attachment — Submitted filename: Response to Reviewers.docx [file pone.0347203.s009.docx]

**Journal**	: PLOS ONE

**Manuscript Number	:** PONE-D-25-37245R4

**Type**	: Research article

**Title**	: A Hybrid CNN-XLSTM-GRU Deep Learning Model with Autoencoder-Based Feature Selection for Hypothyroidism Diagnosis

Dear Editor,

Thank you for noting that the manuscript was reviewed under the title “A Hybrid CNN-XLSTM-GRU Deep Learning Model with Autoencoder-Based Feature Selection for Hypothyroidism Diagnosis.” We thank the reviewer for their recommendations and have revised the manuscript accordingly. Detailed corrections are provided below for each of the reviewer’s comments.

1) The reviewer's comments are highlighted in black colour.

2) Our responses to the comments are highlighted in blue colour.

**Response to Reviewer Comments**

**Comment 1:** Language problems: not detected.

**Response 1:**	The authors thank the reviewer for this kind observation. We are pleased that no language problems were detected. Nevertheless, the manuscript was carefully checked once again to ensure that the presentation remains clear, readable, and consistent throughout.

**Comment 2.1:** Fig 15 - deep blue blocks are not translucent and hide other information - please consider making them a little translucent

**Response 2.1:** The authors thank the reviewer for this helpful observation. Fig. 15 has been revised by reducing the opacity of the deep blue shaded region so that it does not hide the underlying plot elements. This change improves the visual clarity and overall readability of the figure

**Comment 2.2:** Fig 16: The title is not specyfying if presented results were obtained on training or on testing data.

**Response 2.2:** The authors thank the reviewer for this valuable observation. In response, the caption of Fig. 16 was revised to clearly indicate that the reported results were obtained from cross-validation test folds, not from the training data.

**Comment 2.3:** numbering of equation: (8) is improperly alligned (should be: to the right)

**Response 2.3:** The authors thank the reviewer for this careful observation. In response, the alignment of Eq. (8) was corrected, and the equation number is now properly positioned to the right in accordance with the journal formatting style.

**Comment 2.4:** The f1 score is the average (moderate) of recall and precision

**Response 2.4:** The authors thank the reviewer for pointing this out. The notation was corrected in the revised manuscript, and ‘f1’ has been replaced with ‘F1’ to follow standard usage.

**Comment 2.5:** Space is needed between the title of section 3.7 and the preceding paragraph

**Response 2.5:** The authors thank the reviewer for pointing this out. The spacing before the title of Section 3.7 was corrected in the revised manuscript so that it is properly separated from the preceding paragraph.

**Comment 3.1:** Table 10: Authors are using paired t-test. It is not clear if assumptions of t-test were verified (normality of distributions of results and similarity of their variances). This should be verified and discussed within the text. If the assumptions are not met then not parametric test should be used instead of t-test.

**Response 3.1:** The authors thank the reviewer for this important comment. In the revised manuscript, the statistical analysis for Table 10 was clarified. The fold-wise accuracies were first averaged within each repetition, and these repetition-wise mean values were then used for the paired t-test. The normality of the paired differences was checked using the Shapiro–Wilk test and is now reported in the manuscript. Since a paired t-test was used, equal variances between the models were not a required assumption. In addition, the Wilcoxon signed-rank test was included as a non-parametric check, and it gave consistent results.

**Comment 3.2:** Authors write: "These repetition-level mean accuracies were then subjected to the paired ttest thus eliminating the data on individual folds being considered statistically independent observations." This can suggest that results for folds are "considered statistically independent observations".

Do Authors mean "These repetition-level mean accuracies were then subjected to the paired ttest thus eliminating the data on individual folds being considered statistically dependent observations." ?

**Response 3.2:** The authors thank the reviewer for pointing this out. The intended meaning was that the fold-wise results were not treated as statistically independent observations. The sentence has been revised accordingly for clarity.
